# Loss of Genetic Diversity in the Cultured Stocks of the Large Yellow Croaker, *Larimichthys crocea*, Revealed by Microsatellites

**DOI:** 10.3390/ijms13055584

**Published:** 2012-05-09

**Authors:** Le Wang, Xiaofeng Shi, Yongquan Su, Zining Meng, Haoran Lin

**Affiliations:** 1 State Key Laboratory of Biocontrol, Institute of Aquatic Economic Animals and the Guangdong Province Key Laboratory for Aquatic Economic Animals, School of Life Sciences, Sun Yat-sen University, Guangzhou 510275, China; E-Mail: lewang.wang@hotmail.com; 2 College of Ocean & Earth Sciences, Xiamen University, Xiamen 361005, China; E-Mails: xiaofengshi@xmu.edu.cn (X.S.); yqsu@xmu.edu.cn (Y.S.)

**Keywords:** large yellow croaker, *Larimichthys crocea*, genetic diversity, differentiation, microsatellites

## Abstract

The large yellow croaker (*Larimichthys crocea*) is the most important mariculture fish species in China and the wild stocks of this croaker have collapsed in the past decades due to high fishing pressure and habitat degradation. Due to a lack of wild croaker samples, however, studies concerning the genetic changes of the cultured croaker stocks compared to their wild counterparts were never conducted. Here, we collected three wild populations in the northern and central East China Sea during fisheries survey and investigated the differences in terms of genetic diversity and differentiation between and within cultured stocks and wild populations. Our results demonstrated that the cultured croaker had significantly reduced genetic diversity in contrast to the wild populations, and also presented statistically significant differentiation from the wild, indicating that enhancement of the current wild stock should be conducted with caution. These changes may be caused by founder effects, artificial selection and random genetic drift. With a relatively high level of genetic diversity, the wild populations showed important value for improving the ongoing breeding program of this croaker. Further, we detected no differentiation among the wild populations, suggesting that the wild croaker in the northern and central East China Sea should be considered as one unit for management and conservation.

## 1. Introduction

The large yellow croaker, *Larimichthys crocea*, is an economically important marine fish species endemic to China. The production of this croaker reached about 200,000 tons in the mid 1970s and it was once ranked in the top three commercial fish species in mainland China [1,2]. However, the wild resources of this croaker has collapsed in the past decades due to heavy exploitation of spawning and over-wintering aggregations, poor stock management, habitat pollution and climate changes [2–4]. In order to satisfy the needs of consumers for food and also to protect this species from extinction, the Chinese government has conducted successful artificial mariculture for this croaker since 1985 [5]. The aquaculture production reached approximately 70,000 tons in 2006 [6].

However, aquaculture practices are likely to reduce the genetic diversity and further to cause the loss of disease resistance and environmental adaptability, which greatly limit the potential for selective breeding [7–9]. Recently, several biological changes including small size and early age of sexual maturation, low growth rates, poor flesh quality and loss of resistance to disease and cold have been identified in the cultured croaker in contrast to the wild populations [2,6]. Such changes were suggested to be associated with overexploitation of the wild stocks and mariculture operations [2,6]. The decline of quality may be caused by loss of genetic diversity in the cultured stocks [8]. Following successful hatchery production, larvae and fingerlings from hatcheries were released by the Chinese government to restore and enhance the wild stocks of this croaker [10–12]. Such artificial release and random escape from mariculture stocks into the open marine environment can cause potential harmful effects on the genetic make-up of both wild and reared populations without screening the genetic backgrounds of the two types of stocks [7,13,14]. However, no studies have been performed to monitor such genetic make-up and changes between cultured stocks and wild populations of this croaker because of great difficulties in collecting enough wild samples for population genetic studies.

We successfully collected wild populations of the large yellow croaker in fisheries survey from 2007 to 2010, which allowed us to conduct genetic studies as described above. In addition, microsatellite markers have been successfully used in genetic monitoring of the changes between hatchery stocks and wild populations [15–17]. Here, we used 10 microsatellites to analyze the genetic status of both cultured and wild populations of the large yellow croaker in China. The aim of our study was to examine the changes of genetic variation and to assess potential genetic differentiation between cultured and wild populations of this croaker.

## 2. Results

### 2.1. Genetic Variation within Populations

Under exact tests, no consistent deviations from HWE in each sample were detected. After sequential Bonferroni correction, only two locus-population pairs (H37-NB and H54-ZJW) were significant (corrected *P* = 0.0007; Table 1). We also found no evidence of LD among loci. Significant presence of null alleles was detected by Microchecker at one locus, H43. The estimated null allele frequencies in each sample at this locus were more than 0.1 and were statistically significant. As the presence of null alleles may bias the results of genetic variation and particularly differentiation, we therefore excluded this locus from further analysis.

In terms of genetic variation, one locus, H80, was monomorphic in two cultured stocks, DQ and MY (Table 1). The other loci all showed a high level of polymorphism in each sample. The highest A was observed in the wild population YCW (7.899), while the lowest was in cultured stock DQ (4.444). For A_R_, the highest and lowest values were also found in wild population YCW (7.816) and in cultured stock DQ (4.307), respectively. In terms of heterozygosities including H_O_ and H_E_, the highest and the lowest values were also identified in wild population and cultured stock, respectively (Table 1). Further investigation revealed a statistically significant reduction of genetic diversity in the cultured stocks compared to the wild populations. In detail, A_R_ varied from 4.307 (DQ) to 5.037 (NB) with a mean of 4.686 in cultured stocks, which was significantly lower than in wild populations ranging from 5.913 (ZJW) to 7.816 (YCW) with a mean of 6.730 (*P* < 0.01). Similar to A_R_, H_O_ and H_E_ values were also significantly lower in the cultured stocks (from 0.427 to 0.491 and from 0.462 to 0.517 for H_O_ and H_E_, respectively) than in the wild populations (from 0.570 to 0.647 and from 0.591 to 0.649 for H_O_ and H_E_, respectively, *P* < 0.05). However, F_IS_ values showed no significant differences between the cultured stocks and the wild populations (*P* > 0.05). The Bottleneck analysis did not detect signals of recent population reduction for each sample whether in test under TPM or in mode-shift test.

### 2.2. Genetic Differentiation among Populations

**Figure 1:**
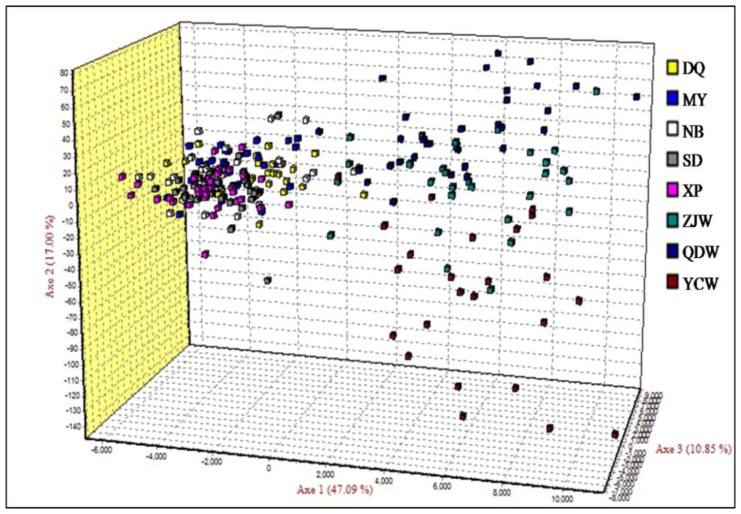
Three dimensional scatter plots for individuals of eight samples of the large yellow croaker based on factor correspondence analysis (FCA) analysis, in which each axis represents one principal factor.

**Figure 2:**
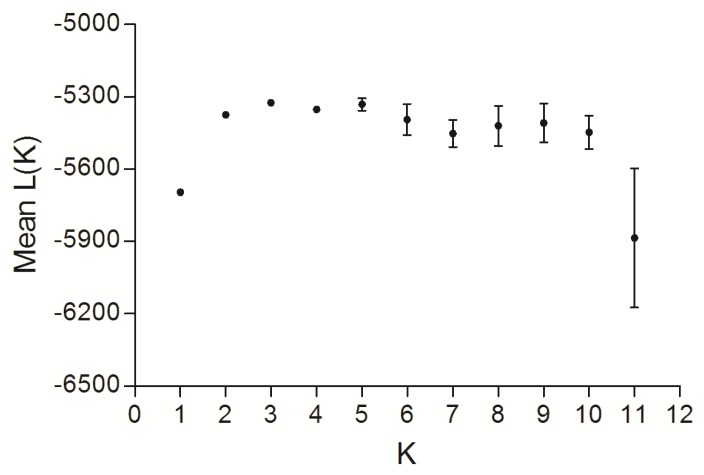
The log likelihood over 20 runs for each *K* values, where the highest value is suggested to be the true number of clusters (*K* = 3).

**Figure 3:**
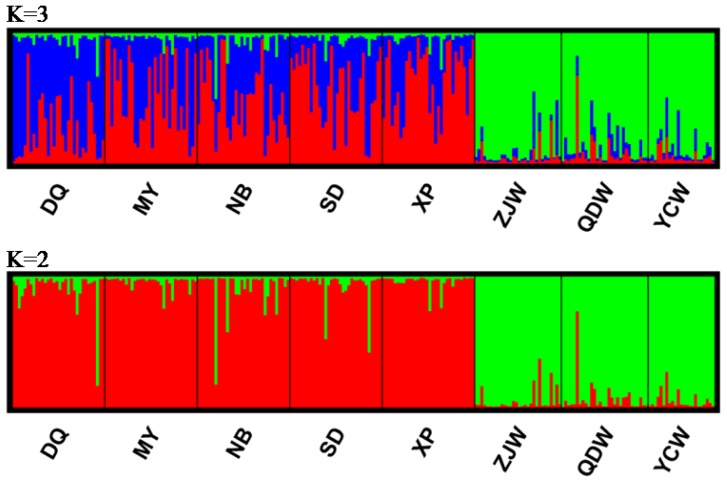
Results of structure analysis based on nine microsatellite loci. Each individual is represented by a vertical line, which is colored according to the assigned groups at estimated *K* = 3 (see Figure 2). Results of *K* = 2 are also presented for identifying the significant differentiation between cultures stocks and wild populations.

The global F_ST_ was 0.070 with 95% confidence interval ranging from 0.023 to 0.173 and was highly significant (*P* < 0.001). Pairwise F_ST_ analysis showed that the wild populations were significantly divergent from the cultured stocks after Bonferroni correction for multiple comparisons (Table 2; *P* < 0.002). Among five cultured stocks, only DQ was significantly different from the others. Interestingly, there was no differentiation detected among the three wild populations (Table 2). Results of AMOVA further supported the significant genetic differentiation between wild populations and cultured stocks, which occupied 10.208% of total genetic variation (*P* < 0.001; Table 3). FCA analysis also revealed significant differences in terms of allele frequency between the cultured stocks and the wild populations (Figure 1). These results were in accordance with the pairwise F_ST_ analysis. In simulations of the Bayesian approach with the program Structure, the mean Ln Likelihood values clearly suggested three clusters as the most likely population structure (Figure 2). The results indicated that almost all the wild individuals were assigned into one cluster, whereas the cultured stocks showed identical genetic properties (Figure 3). However, we observed that the cultured stocks and wild populations were clearly assigned into their own clusters at *K* = 2 (Figure 3). These results strongly supported the results of the other genetic differentiation studies. In addition, we also observed that the DQ stock was slightly divergent from the other cultured stocks both in FCA and in Structure analysis (Figures 1 and 3), which was consistent with F_ST_ results (Table 2).

## 3. Discussion

### 3.1. Microsatellites Polymorphism

Investigation of genetic variation of cultured and wild populations of domesticated animals can provide valuable information for breeding programs and also for conservation genetics [8,18]. In this study, we used polymorphic microsatellites to analyze the genetic differences between the cultured stocks and the wild populations of the large yellow croaker. The allele numbers per locus varied from 3.125 to 16.125 and from 0.000 to 0.889, respectively, which is similar to other marine fish species, such as Atlantic cod (*Gadus morhua*), sea bream (*Pagrus major*) and the orange-spotted grouper (*Epinephelus coioides*), suggesting these microsatellites are sufficient to detect genetic variation in the large yellow croaker [19–21]. Across the eight samples, however, the average allele number and H_E_ per locus were 5.750 and 0.541, respectively, which is much lower than that (20.6 and 0.79 per locus) found in marine fishes [22]. This result might indicate that the large yellow croaker has reduced in genetic diversity due to high fishing pressure and/or artificial breeding.

### 3.2. Genetic Variation within Populations

Aquaculture practices have been broadly reported to have the tendency to reduce genetic variability in cultured stocks of fish species [16,17,23]. Aquaculture practices are detrimental to the domestication process of cultured stocks because a high level of genetic variation is related to adaptive fitness and therefore can provide more chances for organisms to survive under the pressure of artificial and natural selection [24]. In China, the cultured individuals of the large yellow croaker were consistently considered to show a picture of small size, early age of sexual maturation, low growth rates, poor flesh quality and loss of resistance to disease and cold, which may be a reflection of genetic diversity loss [2,6]. As expected, in our study, we detected a statistically significant decline of genetic variation in the cultured stocks compared to their wild counterparts of this croaker (Table 1). The genetic variation measures used in our study involved A_R_, H_O_ and H_E_. Typically, A_R_ is independent of sample size and is more sensitive to be detected in populations of reduced genetic diversity than heterozygosity, as loss of rare alleles shows little effect on heterozygosity [15,18,25]. Here, we found genetic variation declining not only in terms of A_R_ but also in terms of heterozygosity measures, which likely demonstrates that the reduction of genetic variation in the cultured stocks is not caused by sampling and genotyping bias.

The decline of genetic diversity in cultured stocks is typically considered to be the result of interacting founder effects, random genetic drift, and artificial and natural selection in the cultured environments [8,26,27]. In our study, the reduced genetic variation in croaker stocks could be mainly due to founder effects during the domestication process. The broodstocks of this croaker were initially set up in the mid-1980s, when the wild stock of this species had collapsed [2,6]. In this situation, the broodstocks were mainly from random capture of the wild individuals and therefore consisted of only a small number of individuals. This type of broodstock is prone to have great effects on genetic variation of their offspring and ultimately lead to the loss of genetic diversity in the cultured stocks [27]. In addition, random genetic drift and artificial selection much likely played important roles in leading to the loss of genetic diversity in the cultured stocks. On one hand, as an endangered species, there is no large source population available to supplement the small-sized broodstocks of this croaker. In this case, genetic drift is seldom avoided and the cultured stocks would experience excessive loss of genetic variability [8]. On the other hand, these five cultured stocks have experienced artificial breeding for two to three generations. During such domestication process, artificial selection cannot be avoided in order to obtain fingerlings of high quality. Artificial selection has been broadly reported to possibly reduce genetic diversity [18,26,28]. Apart from these factors, natural selection and mutation may also have important effects on genetic diversity [8], though we cannot clearly differentiate whether the two factors reduce or increase the level of genetic diversity in our work. However, it should be noted that all the cultured samples have not experienced bottleneck. This may be due to the short domestication history of this croaker.

### 3.3. Genetic Divergence among Populations

With respect to genetic divergence, pairwise F_ST_, AMOVA, FCA and Structure analysis consistently supported the significant differentiation between cultured stocks and wild populations (Tables 2 and 3, Figures 1 and 3). Such significant differentiation between cultured stocks and wild populations was also observed in many other food fish species, such as salmon (*Salmo salar*), grass carp (*Ctenopharyngodon idella*) and orange-spotted grouper, which was considered to result from artificial selection, founder effects and genetic drift [15,21,29]. Interestingly, we did not detect the signals of differentiation among the three wild populations (Table 2, Figures 1 and 3), suggesting high gene flow in this croaker. It is common for marine fishes, especially for migratory species to present little divergence because of lacking clear barriers to gene flow within open marine environments [30]. This result provides important information for wild stock management and conservation of this croaker. Among the cultured stocks, we observed that only DQ stock was significantly divergent while the others showed little differentiation from each other (Table 2, Figures 1 and 3). Considering the fact that all stocks except for NB experienced selection, the shallow differentiation among cultured stocks might indicate the effects of artificial selection have not been high enough to be detected.

### 3.4. Implications for Artificial Breeding and Conservation

In total, we detected statistically significantly less genetic diversity in the cultured stocks of the large yellow croaker in contrast to their wild counterparts, which was mainly caused by founder effects, random genetic drift and artificial selection involved in aquaculture practices. In addition, significant genetic divergence between cultured stocks and wild populations was also observed. Possessing a high level of genetic variation, the wild croaker showed great value for ongoing selective breeding programs by providing more genetic variation. At the same time, due to the lack of differentiation, the wild populations can be considered as one unit for conservation. However, ongoing artificial wild stock enhancement by releasing of cultured croaker fingerlings should be conducted with caution, as previous studies have suggested that release of hatchery fingerlings with low genetic variation would likely reduce the genetic diversity of the wild populations and further lead to loss of adaptation to variable environments for the wild populations [31–33]. The stock enhancement programs of this croaker were mainly carried out in the north coast of Zhejiang province and more than ten million cultured fries were released from 2000 to 2009, among which about 60,000 were tagged by hanging scutcheon [34]. Recapture study suggested that the released croaker could not only survive but could also spawn in the following years, although the survival rate was quite low [11,34]. Therefore, it is very likely for the released individuals to be integrated into the wild croaker populations. In our study, we detected that the cultured croaker was less diverse in genetic variability than the wild croaker and was significantly divergent from the wild. If the stocked croaker is incorporated into the wild populations, it would definitely change the genetic make-up of the wild croaker populations and further cause adverse effects on the wild croaker.

Since the 1990s, sample collections of the wild populations of this croaker have rarely been reported. In our study, we collected three samples of the wild croaker in the northern and central East China Sea during fisheries survey for several years, which may suggest that this area is “refugia” of this species. Although the wild stock of this croaker has collapsed, we did not detect the signals of recent bottleneck in the three wild croaker populations using microsatellites. In another study, we analyzed the phylogeography of this croaker using the same wild samples as in this study by sequencing mitochondrial *Cytb* and *COI* genes. The results revealed that this species was in the process of population expansion after Pleistocene glaciations, which also suggested few signals of genetic bottleneck in the wild croaker populations [35]. However, previous study has demonstrated that the potential of detecting bottleneck is greatly limited in populations experiencing expansion [36]. Combining the fact that wild croaker was seldom captured, the effective population size of the wild croaker can be rather small. Thus, strict measures by the government, such as reducing fishing pressure and avoiding marine environment pollution in this area must be taken into account to protect this species from extinction.

## 4. Experimental Section

### 4.1. Sample Collection

**Figure 4:**
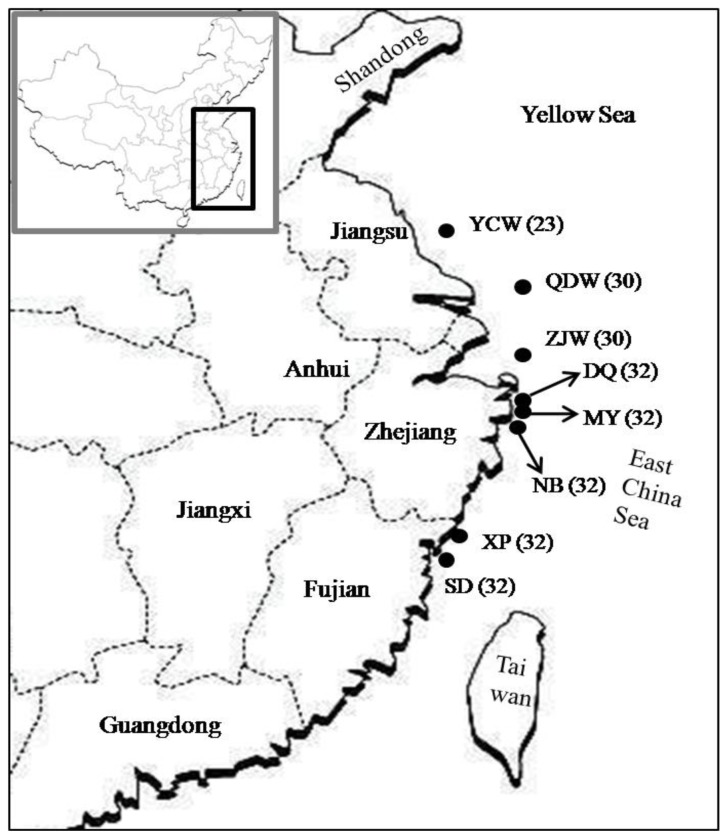
Maps for sampling localities of the large yellow croaker used in this study. YCW: wild population from the northern East China Sea; QDW and ZJW: wild populations from the central East China Sea. DQ, MY, NB: cultured stocks collected from Ningbo, Zhejiang province; XP and SD: cultured stocks from Ningde, Fujian province. Sample size is showed in number after name abbreviation.

We analyzed five cultured stocks and three wild populations of the large yellow croaker. Among these five cultured stocks, three (DQ, MY and NB) were collected from Ningbo, Zhejiang province, while the other two (XP and SD) were from Ningde, Fujian province. In detail, two (MY and NB) and three stocks (DQ, SD and XP) were the third and second generation offspring of the local fish farms, respectively. The broodstocks of these cultured stocks were founded using brooders collected from the central East China Sea. Due to stock collapse in the wild resources of this croaker, however, it is very difficult to capture the wild fish. Fortunately, we collected three wild populations (ZJW, QDW and YCW) from the northern and central East China Sea during several fisheries surveys between 2007 and 2010. Judging from the body size, all the wild individuals were adult. Detailed information about sample size and location is shown in Figure 4. A small piece of muscle tissue or fin clip was collected and stored in 95% ethanol for DNA extraction.

### 4.2. Molecular Methods

Genomic DNA was isolated using the standard phenol-chloroform extraction protocol [37]. All sampled individuals were genotyped using ten microsatellite loci, namely, H16, H31, H33, H37, H43, H47, H54, H65, H80 and H82 [38]. For microsatellite genotyping, forward primers were 5′-labeled with a fluorescent dye HEX or 6-FAM. PCR amplification was performed according to the Molecular Ecology Resources Primer Development Consortium [38]. PCR products were separated on an ABI PRISM 3730 DNA automated sequencer (Applied Biosystems) and were measured according to the ROX-500 standard using GeneMapper (Applied Biosystems).

We employed the number of alleles (A), observed heterozygosities (H_O_), expected heterozygosities (H_E_) and allele riches (A_R_) to measure the genetic variation in each sample. These parameters were calculated in FSTAT version 2.9.3.2 [39]. Exact tests of Hardy-Weinberg equilibrium (HWE) in each sample for each locus and linkage disequilibrium (LD) between pairs of loci were tested using the Markov chain methods implemented in Genepop 4.0 [40]. Inbreeding coefficient (F_IS_) was also estimated using FSTAT version 2.9.3.2 [39]. The genotyping errors and presence of null alleles were checked by using the program Microchecker [41]. The significance of differences in genetic variation between cultured stocks and wild populations were tested using Mann–Whitney *U* test. Recent population size reduction was examined in the form of heterozygote excess using the program Bottleneck version 1.2.02 [42]. The possibility of recent bottlenecks was tested under two-phase model (TPM, with 90% stepwise-mutation) with 1000 iterations and using the graphical mode-shift test by Luikart *et al*. [43].

Genetic differentiation among samples was estimated using pairwise Wright’s F-statistics (F_ST_). The significance was tested by a permutation with 10,000 replicates using ARLEQUIN 3.5 [44] with sequential Bonferroni correction at the significance level of 0.05. Analysis of molecular variance (AMOVA) was performed to partition genetic variance hierarchically between the wild populations and cultured stocks using ARLEQUIN 3.5 [44]. We also performed factor correspondence analysis (FCA) using the program GENETIX 4.05 [45] to detect population structure based on allele frequencies. In addition, a Bayesian method was employed to investigate the population structure of all samples using the program Structure 2.2.3 [46]. This program can estimate the number of putative genetic clusters (K) and assign individuals into corresponding clusters. We conducted this analysis under admixture model and ran for 10^6^ iterations with a burn-in length of 10^6^. The most likely K value was inferred by investigating mean Ln likelihood values.

## 5. Conclusions

In total, our study demonstrated that the cultured croaker had significantly reduced in genetic diversity in contrast to the wild and also presented statistically significant differentiation from the wild, indicating the current wild stock enhancement should be conducted with caution. With a relatively high level of genetic diversity, the wild populations showed important value for the ongoing breeding programs of this croaker. Simultaneously, we detected no differentiation among the wild populations, suggesting that the wild croaker in the northern and central East China Sea should be considered as one unit for management and conservation.

## Figures and Tables

**Table 1:** Summary statistics at nine microsatellite loci across samples of large yellow croaker.

Locus\Saple		DQ	MY	NB	SD	XP	ZJW	QDW	YCW
H16	A	4	3	3	4	3	4	4	5
	A_R_	3.973	3.000	3.000	3.687	3.000	3.995	3.733	4.956
	F_IS_	0.136	0.276	0.116	−0.329	−0.041	−0.004	−0.254	0.054
	H_O_	0.563	0.469	0.531	0.813	0.594	0.633	0.767	0.609
	H_E_	0.649	0.644	0.600	0.615	0.571	0.631	0.614	0.643
H31	A	3	3	4	3	3	4	5	4
	A_R_	3.000	2.992	3.680	2.999	2.998	3.932	4.463	3.999
	F_IS_	−0.136	−0.234	0.082	0.264	−0.191	−0.208	−0.009	−0.080
	H_O_	0.688	0.656	0.500	0.406	0.563	0.767	0.600	0.696
	H_E_	0.607	0.534	0.544	0.550	0.474	0.637	0.595	0.645
H33	A	3	3	3	3	3	3	4	4
	A_R_	2.973	3.000	2.973	3.000	3.000	2.996	3.467	3.913
	F_IS_	−0.254	−0.183	−0.066	0.061	0.157	0.111	−0.059	0.050
	H_O_	0.500	0.531	0.344	0.406	0.438	0.467	0.400	0.435
	H_E_	0.400	0.450	0.323	0.432	0.518	0.524	0.378	0.457
H37	A	7	8	7	9	8	8	8	11
	A_R_	6.976	7.060	6.652	8.776	7.644	7.503	7.658	10.825
	F_IS_	0.151	0.178	0.367	0.165	0.201	0.103	0.083	0.065
	H_O_	0.710	0.656	0.500	0.719	0.625	0.690	0.733	0.783
	H_E_	0.834	0.796	0.785	*	0.859	0.779	0.768	0.799
H47	A	2	3	3	3	3	3	4	4
	A_R_	1.999	2.998	2.998	2.878	2.685	2.467	3.662	3.912
	F_IS_	−0.088	−0.073	0.376	0.376	−0.072	−0.009	−0.074	−0.041
	H_O_	0.188	0.438	0.219	0.094	0.188	0.067	0.233	0.174
	H_E_	0.173	0.408	0.349	0.149	0.175	0.066	0.218	0.167
H54	A	12	15	15	15	13	22	16	21
	A_R_	11.246	13.212	13.117	13.206	11.790	19.669	14.291	21.000
	F_IS_	−0.043	0.141	0.108	−0.054	0.072	0.267	0.160	0.051
	H_O_	0.844	0.781	0.781	0.938	0.813	0.700	0.767	0.909
	H_E_	0.810	0.907	0.874	0.890	0.875	0.950	*	0.910
H65	A	3	3	4	2	2	4	3	4
	A_R_	2.687	2.965	3.660	1.906	1.688	3.916	2.997	3.956
	F_IS_	0.463	0.391	0.555	−0.016	0.000	−0.150	0.137	0.165
	H_O_	0.219	0.125	0.156	0.063	0.031	0.400	0.241	0.391
	H_E_	0.404	0.204	0.348	0.062	0.031	0.349	0.279	0.467
H80	A	1	1	3	2	2	7	5	10
	A_R_	1.000	1.000	2.375	1.688	1.688	6.701	4.968	9.825
	F_IS_	NA	NA	−0.008	0.000	0.000	0.154	0.175	−0.199
	H_O_	NA	NA	0.063	0.031	0.031	0.655	0.556	1.000
	H_E_	NA	NA	0.062	0.031	0.031	0.773	0.672	0.838
H82	A	5	6	7	6	6	7	8	8
	A_R_	4.906	5.985	6.878	5.964	5.963	6.966	7.975	7.955
	F_IS_	−0.079	0.196	0.020	−0.101	−0.066	−0.028	0.030	0.011
	H_O_	0.781	0.625	0.750	0.781	0.750	0.833	0.833	0.826
	H_E_	0.725	0.775	0.765	0.711	0.704	0.811	0.859	0.835
Mean	A	4.444	5.000	5.444	5.222	4.778	6.889	6.333	7.889
	A_R_	4.307	4.690	5.037	4.900	4.495	6.461	5.913	7.816
	F_IS_	0.024	0.094	0.176	0.011	0.031	0.055	0.037	0.004
	H_O_	0.491	0.468	0.427	0.472	0.448	0.579	0.570	0.647
	H_E_	0.503	0.516	0.517	0.478	0.462	0.612	0.591	0.649

* Significant deviation from Hardy–Weinberg equilibrium (HWE) after Bonferroni correction (*P* < 0.0007);

NA, not available.

**Table 2:** Pairwise F_ST_ (below diagonal) and associated *P* values (above diagonal) among cultured stocks and wild populations of the large yellow croaker. Significant *P* values after Bonferroni correction for multiple comparisons are denoted in bold.

Samples	DQ	MY	NB	SD	XP	ZJW	QDW	YCW
DQ	0	**0.000**	**0.001**	**0.000**	**0.000**	**0.000**	**0.000**	**0.000**
MY	0.032	0	0.248	0.008	**0.001**	**0.000**	**0.000**	**0.000**
NB	0.025	0.005	0	0.034	0.039	**0.000**	**0.000**	**0.000**
SD	0.032	0.016	0.012	0	0.095	**0.000**	**0.000**	**0.000**
XP	0.052	0.021	0.012	0.007	0	**0.000**	**0.000**	**0.000**
ZJW	0.119	0.117	0.127	0.134	0.143	0	0.048	0.011
QDW	0.107	0.104	0.105	0.121	0.138	0.009	0	0.049
YCW	0.096	0.096	0.092	0.100	0.112	0.015	0.010	0

**Table 3:** Results of analysis of molecular variance (AMOVA) between cultured stocks and wild populations of the large yellow croaker.

Source of Variation	Sum of Squares	Variance Components	Percentage Variation	*P* Value
Among groups	64.648	0.280	10.208	0.000
Among populations within groups	31.072	0.043	1.576	0.000
Among individuals within populations	597.807	0.133	4.853	0.000
Within individuals	554.000	2.287	83.363	0.000
Total	1247.526	2.743	100.000	
